# Brentuximab vedotin in treating Chinese patients with lymphoma: A multicenter, real‐world study

**DOI:** 10.1002/cam4.6733

**Published:** 2023-11-17

**Authors:** Xudong Zhang, Honghan Qiao, Xiaofei Chai, Xue Gao, Rongjun Ma, Yufu Li, Zunmin Zhu, Mingzhi Zhang

**Affiliations:** ^1^ The First Affiliated Hospital of Zhengzhou University ZhengZhou China; ^2^ Henan Cancer Hospital ZhengZhou China; ^3^ Henan Provincial People's Hospital ZhengZhou China

**Keywords:** brentuximab vedotin, effectiveness, lymphoma, real‐world study, safety

## Abstract

**Background:**

Brentuximab vedotin (BV) was approved as a therapy for patients with CD30‐positive lymphoma in China in 2020 based on the results of multiple clinical trails. Few Chinese real‐world data of its use are yet available. Herein, we present the application situation of BV in patients with lymphoma among different hospitals in Henan province in China under real‐world conditions.

**Methods:**

This was a multicenter and retrospective study in 104 patients with lymphoma who received BV for the first time between August 2020 and September 2022 across eight centers in Henan province. Data on the clinical use, effectiveness and adverse events (AEs) of BV were extracted from patient medical records. Short‐term effectiveness was assessed based on objective response rate (ORR), complete response (CR), partial response (PR), stable disease (SD) and progressive disease (PD). Progression‐free survival (PFS) and overall survival (OS) were calculated using Kaplan–Meier method. Safety was also evaluated in our study.

**Results:**

104 lymphoma patients were enrolled in our study, who had completed a median of two cycles (range,1–8) of BV‐based treatment. A total of 72.1% of patients were relapsed/refractory (R/R) lymphoma, and only 27.9% were previously untreated lymphoma who received BV in frontline treatment settings. Among them who received effectiveness evaluation, the ORR achieved 64.5% (CR 25.8%, PR 38.7%). After a median follow‐up of 11 months, the 6‐month PFS rate and OS rate achieved 77.2% and 90.1% respectively, and the 12‐month PFS rate and OS rate achieved 77.2% and 79.9% respectively. In general, BV‐based treatment was well‐tolerated, with 38.5% incidence of grade ≥3 AEs. The most commonly reported AEs were hematologic disorders, especially neutropenia, with the incidence reaching 50.0%.

**Conclusions:**

BV‐based regimens could be a promising therapeutic option with remarkable effectiveness and moderate toxicity in treating Chinese lymphoma patients with CD30 expression.

## 1 INTRODUCTION

Lymphomas are a highly heterogeneous group of malignancies arising from the hematopoietic system, and in recent years, the incidence of lymphomas has shown an increasing trend in China. The progression of biomarker detection and the explosion of novel targeted drugs have significantly complemented the deficiency of traditional treatment in lymphoma.

CD30, a transmembrane glycoprotein receptor, is mainly expressed on the surface of activated B cells, T cells, and natural killer cells in healthy tissue.^1^ In lymphoma, CD30, as an important diagnostic biomarker, is universally expressed in classical Hodgkin lymphoma (cHL) and anaplastic large cell lymphoma (ALCL), but in other lymphoproliferative disorders, such as diffuse large B‐cell lymphoma (DLBCL), peripheral T‐cell lymphoma (PTCL) and cutaneous T‐cell lymphoma (CTCL), it is also expressed to variable degrees.^2^ Due to its high expression on malignant cells and limited expression on normal cells, CD30 is regarded as an ideal therapeutic target in lymphoma.^3^

Brentuximab vedotin (BV) is an antibody–drug conjugate (ADC) targeting CD30.^4^ In previous clinical trials conducted in Western patients, BV has demonstrated considerable effectiveness and safety in the treatment of lymphoma.^5^, ^6^, ^7^ Therefore, BV was initially approved for treating cHL and systemic anaplastic large cell lymphoma (sALCL) by the Food and Drug Administration (FDA) in the USA in 2011. In May 2020, BV was officially authorized for the treatment of adult CD30‐positive lymphoma by the National Medicinal Products Administration (NMPA) in China. Real‐world data on BV use and evidence regarding its effectiveness and safety in clinical practice, however, remain lacking.

In this multicenter and retrospective study, we aimed to understand the current state of BV application in a relatively large lymphoma patient population in China and to evaluate the effectiveness and safety of BV in Chinese patients in real‐world settings within 2 years after it was formally approved. This real‐world study was designed to lay a solid foundation for better clinical use of BV in the Chinese population.

## 2 MATERIALS AND METHODS

### 2.1 Study design

This was a multicenter and retrospective study performed at eight centers in Henan Province in China. Patients with lymphoma who initially received BV treatment between August 2020 and September 2022 were included in the study. Patients received BV 1.8 mg/kg intravenously on Day 1 of each 3‐week cycle, and dose reduction to 1.2 mg/kg was allowed depending on the lymphoma type and toxicity. Regimens could be continued until disease progression or the occurrence of unacceptable AEs. Data were collected from patient medical records from all centers and unified in a final database. The study was conducted at the First Affiliated Hospital of Zhengzhou University and was approved by the ethics committee of each participating center. All enrolled patients signed consent for targeted drug therapy before starting treatment.

### 2.2 Patient

**FIGURE 1:**
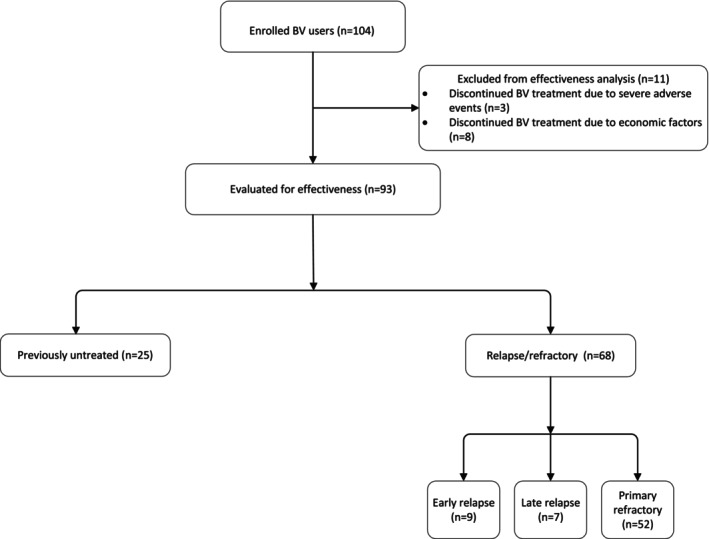
Procedures of patients selections.

Our study involved patients diagnosed with lymphoma confirmed by pathology who received CD30 detection by immunohistochemistry (IHC) prior to BV treatment and were treated with ≥1 dose of BV. A total of 104 eligible patients were included, excluding those who had incomplete medical records or missing follow‐up information. Clinical characteristics of these patients, such as age, sex, hospital, lymphoma subtypes, Eastern Cooperative Oncology Group (ECOG) score, disease stage, B symptoms, CD30 expression, bone marrow involvement, central involvement, previous treatment regimens, disease response, and adverse events (AEs), were collected for further analysis. Eleven patients who discontinued BV treatment due to severe AEs or economic factors were excluded from the effectiveness assessment, and 93 patients were ultimately included in the effectiveness analysis. Procedures of patient selection are displayed in Figure 1.

### 2.3 Assessment of effectiveness and AEs

Response evaluation was conducted using computed tomography and positron emission computed tomography (PET‐CT). Response assessment was categorized as complete response (CR), partial response (PR), stable disease (SD) or progressive disease (PD).^8^ Objective response rate (ORR) was defined as the proportion of patients with CR and PR. Progression‐free survival (PFS) was defined from the initiation of BV‐based therapy to first relapse, progression or death and the date of the last follow‐up. Overall survival (OS) was defined as that from the date of starting BV to death or last observation for any cause. Safety and tolerability were assessed during the observation period, and AEs were recorded and graded according to the National Cancer Institute Common Terminology Criteria for Adverse Events (CTCAE version 4.0).

### 2.4 Follow‐up and statistical analysis

Follow‐up was extended via telephone interviews or further consultation until December 2022. Categorical variables are presented as numbers and percentages. Continuous variables are expressed as median and range. Effectiveness was assessed using the chi‐squared test. OS and PFS were determined using the Kaplan–Meier methodology, and differences between groups were compared using the log‐rank test. Statistical analyses of our study were conducted using SPSS software version 26.0 and GraphPad Prism version 8. A *p* value of <0.05 was considered statistically significant.

## 3 RESULTS

### 3.1 Baseline characteristics

A total of 104 patients diagnosed with lymphoma were enrolled from eight centers in Henan Province, and 93 patients were included in the effectiveness analysis. The baseline characteristics of these patients are shown in Table 1. The median age prior to BV treatment was 51 years (range, 2–88 years). A total of 30.8% of patients were over the age of 60, and 53.9% of patients were male. The most common lymphoma type was sALCL (29.8%), followed by cHL (27.9%). Other lymphoma types, including AITL, PTCL‐NOS, DLBCL, ENKTCL, MF, HSTCL, and pcALCL, occupied a total proportion of 42.3%. The majority of patients were identified with Ann Arbor stage III (20.2%) and IV (61.5%), and 11 (10.6%) patients had B symptoms (fever, drenching night sweats and loss of >10% of body weight over 6 months) before receiving BV. The ECOG score was <2 in half of the patients at the time of BV treatment. Only 2 (1.9%) patients were reported to have no CD30 expression by IHC detection. Sixteen (15.4%) patients developed bone marrow involvement, and four (3.9%) patients had central involvement.

**TABLE 1:** Baseline characteristics of patients treated with brentuximab vedotin.

Characteristics	*N* (%)
Patients	104 (100.0%)
Age, years	
Median age (range)	51 (2–88)
>60 years	32 (30.8%)
≤60 years	72 (69.2%)
Sex	
Male	56 (53.9%)
Female	48 (46.2%)
Center	
The First Affiliated Hospital of Zhengzhou University	36 (34.6%)
Henan Cancer Hospital	34 (32.7%)
Henan Provincial People's Hospital	26 (25.0%)
Anyang People's Hospital	3 (2.9%)
Zhengzhou Central Hospital	2 (1.9%)
The Second Affiliated Hospital of Zhengzhou University	1 (1.0%)
The Sixth People's Hospital of Zhengzhou	1 (1.0%)
The First Affiliated Hospital of Xinxiang Medical University	1 (1.0%)
Lymphoma type	
sALCL	31 (29.8%)
cHL	29 (27.9%)
AITL	12 (11.5%)
PTCL‐NOS	13 (12.5%)
DLBCL	9 (8.7%)
ENKTCL	5 (4.8%)
MF	3 (2.9%)
pcALCL	1 (1.0%)
HSTCL	1 (1.0%)
Stage	
I	1 (1.0%)
II	18 (17.3%)
III	21 (20.2%)
IV	64 (61.5%)
B symptoms	
Yes	11 (10.6%)
No	93 (89.4%)
ECOG	
<2	52 (50.0%)
≥2	52 (50.0%)
CD30 expression	
Positive	102 (98.1%)
Negative	2 (1.9%)
Bone marrow involvement	
Yes	16 (15.4%)
No	88 (84.6%)
Central marrow involvement	
Yes	4 (3.9%)
No	100 (96.1%)
Disease status prior to BV	
Untreated	29 (27.9%)
Relapsed	19 (18.3%)
Refractory	56 (53.8%)
Prior systemic treatment cycles, *n*	
Median	6
Range	0–28
Prior ASCT	
Yes	3 (2.9%)
No	101 (97.1%)
BV cycles, *n*	
Median	2
Range	1–8

All patients completed ≥1 cycle of BV‐based therapy, and only 27.9% of them were newly diagnosed without any prior therapy. The median number of prior therapies was 6 (range, 0–28), and 53.6% were refractory to their previous treatment. Three (2.9%) patients underwent autologous stem cell transplantation (ASCT) prior to BV. The median cycle number of BV therapies was 2 (range, 1–8), which was relatively limited.

### 3.2 Treatment regimens

BV‐based treatment in different types of lymphoma is displayed in Table 2. In 29 HL patients, 7 were newly diagnosed in the advanced stage and most commonly received BV combined with modified chemotherapy regimens of doxorubicin, vinblastine, and dacarbazine (AVD) as frontline treatment. For treating 22 relapsed/refractory (R/R) HL patients, BV combined with chemotherapy (AVD regimen or bendamustine) was the main alternative as a second‐ or later‐line therapy compared with BV in combination with immunotherapy or monotherapy. For BV in NHL, differences could be observed in patients with T‐cell lymphoma (PTCL and CTCL) and B‐cell non‐Hodgkin lymphoma. In DLBCL, BV with monoclonal antibody rituximab was used as frontline therapy, while in TCL, the most common combinations as first‐line therapies were BV and chemotherapy, especially with cyclophosphamide, doxorubicin and prednisone (CHP). For relapsed/refractory NHL, most patients were exposed to BV with chemotherapy, especially a CHP‐like regimen, and the minority chose BV plus immune checkpoint inhibitors or monotherapy. Other targeted agents, such as Bruton's tyrosine kinase inhibitor (BTKi) and B‐cell lymphoma‐2 inhibitor (BCL‐2i), were also used for R/R NHL patients.

**TABLE 2:** Treatment regimens in different types of lymphoma

Lymphoma type			Disease status prior to BV	Treatment		Patients, *n*
HL				Combined therapies with BV	Regimens	
				Chemotherapy	AVD	6
			Initial treatment	Immunotherapy	PD‐1	1
			Relapsed/refractory	Chemotherapy	AVD	5
					Bendamustin	4
					Other	3
				Immunotherapy	PD‐1	7
				No		3
NHL	BCL		Initial treatment	Chemotherapy	R‐CHP	1
			Relapsed/refractory	Chemotherapy	CHP	2
					Other	1
				Immunotherapy	PD‐1	2
				Targeted regimens	BTK inhibitor	2
				No		1
	TCL	PTCL	Initial treatment	Chemotherapy	CHP	18
					Other	1
			Relapsed/refractory	Chemotherapy	CHP	10
					Gemox	5
					Other	14
				Immunotherapy	PD‐1	2
				Targeted regimens	Venetoclax	1
					TK inhibitor	5
					Selinexor	1
				No		5
		CTCL	Initial treatment	Chemotherapy	CHOPE	1
					Other	1
			Relapsed/refractory	Chemotherapy	Decitabine	1
				Immunotherapy	PD‐1	1

### 3.3 Response to treatment

At a median follow‐up of 11 months for the entire cohort, response was assessed in 93 of 104 eligible patients. The total ORR among them was 64.5% (CR: 25.8%, PR: 38.7%). In the cHL group, 9 patients achieved CR, and 10 achieved PR; the ORR was 70.4%. In the ALCL group, the ORR reached 69.0% (11 CRs, 9 PRs). In other types of lymphoma, over half of the group (56.8%) achieved an objective response, including 4 CRs and 17 PRs (Table 3). No significant differences in ORR were observed among patients with different lymphoma types in the three groups (*p* = 0.44). Twenty‐five previously untreated patients and 68 R/R patients were involved in the response assessment in our study. The R/R patients included 9 with early relapse (remission duration ≤1 year), 7 with late relapse (remission duration>1 year), and 52 with primary refractory disease (27 achieved PR/SD to front‐line therapy and 25 achieved PD to front‐line therapy). The ORR reached 66.7%, 28.6%, and 63.5%, respectively.

**TABLE 3:** Response to treatment in different lymphoma types.

Effectiveness, *n* (%)	Total, *n* = 93	cHL, *n* = 27	ALCL, *n* = 29	Other types*, *n* = 37
ORR	60 (64.5)	19 (70.4)	20 (69.0)	21 (56.8)
CR	24 (25.8)	9 (33.3)	11 (37.9)	4 (10.8)
PR	36 (38.7)	10 (37.0)	9 (31.0)	17 (45.9)
SD	12 (12.9)	4 (14.8)	3 (10.3)	5 (13.5)
PD	21 (22.6)	4 (14.8)	6 (20.7)	11 (29.7)

Other types * including AITL, PTCL‐NOS, DLBCL, MF, ENKTCL, HSTCL.

### 3.4 Survival outcomes

**FIGURE 2:**
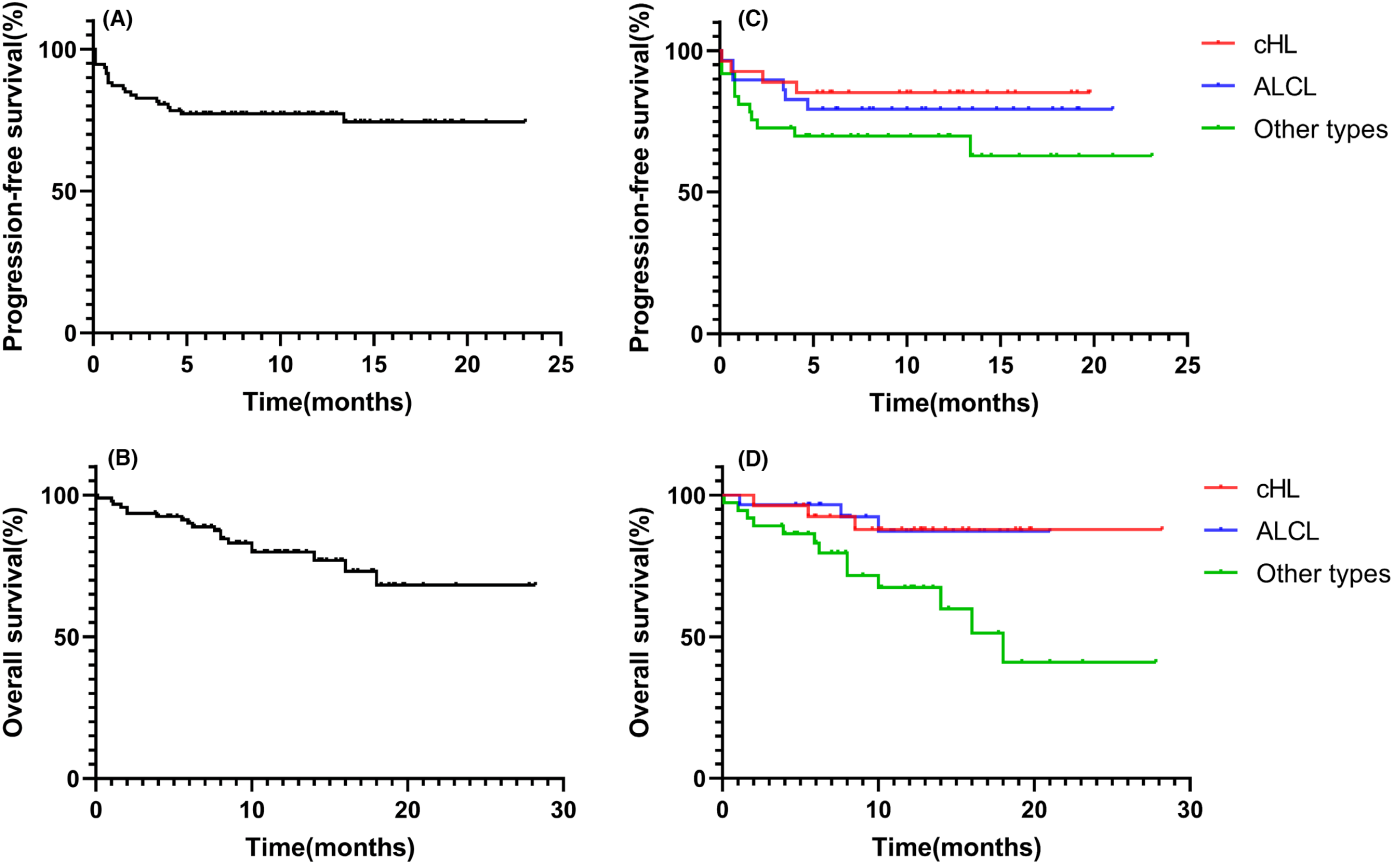
Kaplan–Meier survival curves of BV‐based treatment in 93 lymphoma patients. (A) The progression‐free survival (PFS) curve among 93 patients. (B) The overall survival (OS) curve among 93 patients. (C) The PFS curve among patients stratified according to lymphoma subtypes. (D) The OS curve among patients stratified according to lymphoma subtypes.

A total of 93 patients were available for survival analysis. After a median follow‐up time of 11 (range: 0.1–27.8) months, 19 patients died, and 22 had disease progression. Kaplan–Meier survival curves for PFS and OS are shown in Figure 2. The 6‐ and 12‐month PFS rates were 77.2% and 77.2%, respectively, and the 6‐ and 12‐month OS rates were 90.1% and 79.9%, respectively. When considering the survival outcomes of different subtypes of lymphoma, the 6‐ and 12‐month PFS rates of cHL, ALCL and other lymphoma types were 85.2%, 79.3%, and 69.9%, respectively, with 6‐month OS rates of 92.4%, 96.6%, and 83.1% and 12‐month OS rates of 87.8%, 87.2%, and 67.4% for cHL, ALCL and other lymphoma types, respectively. The median PFS and the median survival time were not reached for the entire cohort. No significant differences in PFS were noted between patients in the three groups (*p* = 0.21). The OS, however, was significantly greater in patients with cHL and ALCL than in those with other lymphoma types (*p* = 0.021 and 0.018).

### 3.5 Safety

All patients who had completed at least one cycle of BV‐based treatment were included in the safety analysis. A total of 98 patients developed AEs of any grade, and the total incidence was 94.2%; 40 patients developed grade ≥3 AEs, and the incidence was 38.5%. The most commonly reported AEs were hematologic abnormalities (64.4%), infection (31.7%) and gastrointestinal reactions (13.5%). The hematologic AEs occurring in patients included neutropenia, anemia and thrombocytopenia, and the most frequent type was neutropenia (50.0%). Additionally, 11 of 104 patients (10.6%) developed peripheral neuropathy (PN), which was moderate and reversible. Neutropenia was the most common Grade 3 or higher AE, presenting in 18 of 40 patients (45.0%). Generally, the AEs of BV‐based therapy were mostly tolerable, while only five patients discontinued BV treatment, and four experienced dose adjustment because of severe AEs. AEs occuring during BV‐based treatment are illustrated in Table 4.

**TABLE 4:** AEs during BV‐based treatment.

AEs	No. of patients, *n* (%) any grade	Grade ≥3
**Hematologic disorder**		
Neutropenia	52 (50.0%)	18 (17.3%)
Anemia	36 (34.6%)	15 (14.4%)
Thrombocytopenia	26 (25.0%)	16 (15.4%)
Infection	33 (31.7%)	11 (10.6%)
**Gastrointestinal reactions**		
Nausea	9 (8.7%)	0
Diarrhea	3 (2.9%)	0
Constipation	1 (1.0%)	0
**Peripheral neuropathy**	11 (10.6%)	3 (2.9%)
**Fever**	11 (10.6%)	6 (5.8%)
**Abnormal liver function**	8 (7.7%)	1 (1.0%)
**Serum chemistry abnormalities**	5 (4.8%)	0
**Rash**	3 (2.9%)	0

## 4 DISCUSSION

BV has shown promising results in Western countries since its launch, and it has been 2 years since BV belatedly arrived in China to treat CD30‐positive lymphomas. Chinese expert consensus on principles for the use of BV in clinical practice was published recently in 2022. Previously, a Phase II single‐arm study in Chinese patients with R/R cHL or ALCL confirmed BV as a potential treatment option with an ORR of 69%, a median PFS of 12.1 months and a median OS of 13.5 months.^9^ Real‐world studies with large patient populations, long follow‐up times and fewer restrictions on enrollment could fill the data gap between clinical trials and real‐world settings. However, data on the current clinical application of BV in the real world in China have yet to be supplemented. Thus, we conducted a retrospective study collecting real‐world data of all BV users between August 2020 and September 2022 from eight institutions in Henan Province to introduce the current use of BV in the Chinese lymphoma population and to develop a more comprehensive understanding of its effectiveness and safety in complex real‐world clinical practice.

In our study, BV demonstrated dramatic treatment outcomes, especially in cHL and ALCL patients, with ORRs of 70.4% and 69.0%, 12‐month PFS rates of 85.2% and 79.3% and 12‐month OS rates of 87.8% and 87.2%, respectively. The 12‐month PFS and OS rates in R/R cHL patients were 81.8% and 84.6%, respectively, and the 12‐month PFS and OS rates in R/R ALCL patients were 83.3% and 81.4%, respectively, which were generally better than the survival outcomes of BV monotherapy revealed by the Chinese clinical trial,^9^ possibly owing to the combination of BV and other agents. In the cHL group, six untreated patients with advanced‐stage disease received the A‐AVD regimen as frontline therapy, with an overall CR rate of 80.0% (1 was excluded from the effectiveness assessment, and 1 achieved SD). Compared to ABVD treatment during the same period (CR 73.0%), BV showed a clinical advantage when applied earlier, which was consistent with the results of previous studies.^10^ In the ALCL group, 11 of 12 untreated patients received the A‐CHP regimen as frontline treatment, among whom three experienced disease progression and subsequently turned to second‐line treatment. Considering the small sample size, short follow‐up time, poor treatment continuity and the influence of the COVID‐19 pandemic in the real world, our evidence needs to be validated in comparison to a 5‐year ECHELON‐2 study (5‐year PFS rates were 51.4% in the A + CHP group and 43.0% in the CHOP group).^11^ Moreover, the use of BV has also been expanded to other lymphomas, such as other peripheral T‐cell lymphomas and B‐cell non‐Hodgkin lymphomas, and our results suggest that BV might be a promising option for CD30‐positive lymphomas apart from cHL and ALCL, with the ORR reaching 56.8%, the 6‐month PFS rate reaching 69.9% and the 6‐month OS rate reaching 83.1%, supporting the conclusions from clinical trials.^12^

In general, the AEs reported during BV‐based treatment were acceptable in our cohort. The incidence of AEs emerging in patients who received BV monotherapy was 88.9%, and the incidence of AEs emerging in those who received BV combined therapy was 94.7%. The most commonly developed AEs and grade ≥3 AEs were both hematologic toxicities, especially neutropenia. However, the incidence of peripheral neuropathy was not as high as that reported in Western and Chinese clinical trials before,^6^, ^9^, ^13^ and the most commonly developed symptom of PN was numbness. Interestingly, in real‐world clinical practice, PN seemed to be less frequent and generally mild in terms of severity,^14^ which could be attributed to physicians paying great attention to its prevention by enhancing the protection and nutrition of nerves and avoiding the application of other neurotoxic drugs.

A total of 104 patients received a median of two cycles of BV treatment, and 23 patients only used BV once, which may affect disease control. The major cause of this relatively low number of BV cycles is economic factors. It was estimated, in our study, that nearly 20% of patients claimed to refuse or stop BV‐based therapies due to financial burdens. Nevertheless, one analysis concluded that BV might be a cost‐effective therapy in comparison to traditional chemotherapy in treating R/R sALCL from a Chinese health care perspective.^15^ In the future, we are looking forward to the drop in BV prices in China, which will bring significant benefits to lymphoma patients.

Overall, 14 relapsed/refractory lymphoma patients underwent autologous hematopoietic stem cell transplantation (ASCT). Among them, three patients received BV‐based salvage treatment after ASCT failure, whereas 10 patients received BV as a bridging therapy prior to ASCT. For patients relapsing after ASCT, two reached the best response with CR to BV‐based salvage regimens, and clinical outcomes were improved in patients receiving BV as the first‐line treatment after post‐ASCT relapse, which was in line with the results of several real‐world studies.^16^, ^17^ For 10 patients who were initially considered ineligible for HSCT due to insufficient response to previous treatment, BV enabled nine to achieve objective responses (five PRs and four CRs) and successfully bridge to ASCT, confirming its role in inducing remissions as an efficacious bridge to transplant in R/R lymphoma patients. However, patients who benefited from this treatment were in a limited range, and BV as a bridge therapy still has great potential in real world applications.

Notably, one sALCL patient with a history of two cycles of BV combined with decitabine had experienced a PR but discontinued treatment because of the pandemic and subsequently had disease progression and was retreated with BV in combination with mitoxantrone liposomes and selinexor at intervals of 3 months. After one dose of BV, this patient rapidly achieved an objective response with mild toxicity. This result exhibits our attempt at BV retreatment in pretreated lymphoma patients who initially responded to BV on the basis of previous studies suggesting its efficacy and similar safety profiles during retreatment.^18^, ^19^ Nevertheless, in our patients, one with ALK+sALCL developed an SD after one BV and later received the CHOPE regimen before ASCT, then used two cycles of BV plus alectinib for retreatment after ASCT failure and soon reached a CR, which indicated the feasibility of BV retreatment in patients who relapsed or progressed after frontline BV treatment.

Questions concerning the relationship between CD30 expression level and the clinical response to BV treatment remain unclear. It is also quite controversial whether to allow CD30‐negative patients to receive the CD30‐directed drug BV. One issue with CD30‐ mycosis fungoides from Beijing University Hospital was reported to achieve an outstanding response to BV.^20^ Our study included two patients diagnosed with refractory T‐cell lymphoma with CD30 expression that was undetectable by IHC, and both had disease progression after one or two cycles of BV‐based therapy. Compared with the impressive results of five prospective clinical studies in NHL,^21^ our patients manifested a dismal response to BV. Therefore, based on our results, we concluded that no expression of CD30 indicates a poor prognosis for patients receiving CD30‐targeted treatment regimens. Frankly, in our study, only 6 cases out of 104 were described as quantitative patterns of CD30 expression after IHC detection, restricting our exploration of the correlation between response and CD30 expression. This finding also reflects the current status of CD30 detection and interpretation in China: most results of CD30 IHC detection were simply reported as qualitative descriptions rather than quantitative descriptions in real‐world settings. Nevertheless, many recent studies have confirmed that the level of CD30 expression may not be clearly correlated with the extent of clinical response to BV treatment in various types of lymphoma.^22^, ^23^ The possible factors are as follows. First, although IHC is most commonly used for CD30 detection in clinical practice, the accuracy and sensitivity of IHC remain limited, contributing to the nondetection of very low levels of CD30 expression.^24^ IHC detection methods vary from procedures for tissue preparation and staining processes to CD30 antibodies and staining platforms used among different centers under real‐world conditions, which may impede accurate diagnosis and therapeutic decision‐making. Second, CD30‐independent mechanisms of action, including the bystander effect, antibody‐dependent cellular phagocytosis, immunogenic cell death (ICD), and depletion of CD30‐positive T‐regulatory cells,^25^, ^26^, ^27^ would consequently result in clinical benefits of BV treatment in CD30‐negative patients.

In conclusion, this multicenter, real‐world study on BV‐based treatment enrolled a relatively large lymphoma population in China. The limitations of our research mainly lie in the short follow‐up time and less detailed grouping. Future efforts should be made to extend the follow‐up time in multiple centers for continuous application to explore more latent possibilities of BV‐based treatment in clinical practice.

## AUTHOR CONTRIBUTIONS

**Xudong Zhang:** Conceptualization (equal); funding acquisition (equal); project administration (equal); writing – review and editing (equal). **Honghan Qiao:** Data curation (equal); formal analysis (equal); writing – original draft (equal). **Xiaofei Chai:** Data curation (equal); formal analysis (equal). **Xue Gao:** Data curation (equal). **Rongjun Ma:** Data curation (equal). **Yufu Li:** Project administration (equal); supervision (equal). **Zunmin Zhu:** Project administration (equal); supervision (equal). **Mingzhi Zhang:** Funding acquisition (equal); project administration (equal); supervision (equal).

## FUNDING INFORMATION

The study was supported by the National Natural Science Foundation of China (No. 82070210, 81970184, and 82170183).

## CONFLICT OF INTEREST STATEMENT

The authors declare no potential competing interests.

## ETHICS STATEMENT

The study was approved by the Clinical and Research Ethics Committee of the First Affiliated Hospital of Zhengzhou University, Zhengzhou, China (2022‐KY‐0869‐001). Consent to participate and to public was not required for this study.

## Data Availability

The original contributions presented in this study are included in the article, further inquiries can be directed to the corresponding author.

## References

[1] Dürkop H , Foss HD , Eitelbach F , et al. Expression of the CD30 antigen in non‐lymphoid tissues and cells. J Pathol. 2000;190(5):613‐618. 10727988 10.1002/(SICI)1096-9896(200004)190:5<613::AID-PATH559>3.0.CO;2-0

[2] van der Weyden CA , Pileri SA , Feldman AL , Whisstock J , Prince HM . Understanding CD30 biology and therapeutic targeting: a historical perspective providing insight into future directions. Blood Cancer J. 2017;7(9):e603. 28885612 10.1038/bcj.2017.85 PMC5709754

[3] Schirrmann T , Steinwand M , Wezler X , Ten Haaf A , Tur MK , Barth S . CD30 as a therapeutic target for lymphoma. BioDrugs. 2014;28(2):181‐209. 24043362 10.1007/s40259-013-0068-8

[4] Li WQ , Guo HF , Li LY , Zhang YF , Cui JW . The promising role of antibody drug conjugate in cancer therapy: combining targeting ability with cytotoxicity effectively. Cancer Med. 2021;10(14):4677‐4696. 34165267 10.1002/cam4.4052 PMC8290258

[5] Chen R , Gopal AK , Smith SE , et al. Five‐year survival and durability results of brentuximab vedotin in patients with relapsed or refractory Hodgkin lymphoma. Blood. 2016;128(12):1562‐1566. 27432875 10.1182/blood-2016-02-699850 PMC5034737

[6] Younes A , Gopal AK , Smith SE , et al. Results of a pivotal phase II study of brentuximab vedotin for patients with relapsed or refractory Hodgkin's lymphoma. J Clin Oncol. 2012;30(18):2183‐2189. 22454421 10.1200/JCO.2011.38.0410 PMC3646316

[7] Pro B , Advani R , Brice P , et al. Five‐year results of brentuximab vedotin in patients with relapsed or refractory systemic anaplastic large cell lymphoma. Blood. 2017;130(25):2709‐2717. 28974506 10.1182/blood-2017-05-780049 PMC5746164

[8] Younes A , Hilden P , Coiffier B , et al. International Working Group consensus response evaluation criteria in lymphoma (RECIL 2017). Ann Oncol. 2017;28(7):1436‐1447. 28379322 10.1093/annonc/mdx097 PMC5834038

[9] Song Y , Guo Y , Huang H , et al. Phase II single‐arm study of brentuximab vedotin in Chinese patients with relapsed/refractory classical Hodgkin lymphoma or systemic anaplastic large cell lymphoma. Expert Rev Hematol. 2021;14(9):867‐875. 34275403 10.1080/17474086.2021.1942831

[10] Ansell SM , Radford J , Connors JM , et al. Overall survival with brentuximab vedotin in stage III or IV Hodgkin's lymphoma. N Engl J Med. 2022;387(4):310‐320. 35830649 10.1056/NEJMoa2206125

[11] Horwitz S , O'Connor OA , Pro B , et al. The ECHELON‐2 trial: 5‐year results of a randomized, phase III study of brentuximab vedotin with chemotherapy for CD30‐positive peripheral T‐cell lymphoma. Ann Oncol. 2022;33(3):288‐298. 34921960 10.1016/j.annonc.2021.12.002 PMC9447792

[12] Kim SJ , Yoon DH , Kim JS , et al. Efficacy of brentuximab vedotin in relapsed or refractory high‐CD30‐expressing non‐Hodgkin lymphomas: results of a multicenter, open‐labeled phase II trial. Cancer Res Treat. 2020;52(2):374‐387. 31476851 10.4143/crt.2019.198 PMC7176958

[13] Gao S , Zhang M , Wu K , et al. Risk of adverse events in lymphoma patients treated with brentuximab vedotin: a systematic review and meta‐analysis. Expert Opin Drug Saf. 2020;19(5):617‐623. 31955620 10.1080/14740338.2020.1718103

[14] Plattel WJ , Bergamasco A , Trinchese F , et al. Effectiveness of brentuximab vedotin monotherapy in relapsed or refractory Hodgkin lymphoma: a systematic review and meta‐analysis. Leuk Lymphoma. 2021;62(14):3320‐3332. 34323643 10.1080/10428194.2021.1957865

[15] Liu J , Zheng L , Chuang LH . Cost‐effectiveness of brentuximab vedotin for relapsed or refractory systemic anaplastic large‐cell lymphoma in China. J Med Econ. 2022;25(1):99‐107. 34927526 10.1080/13696998.2021.2020567

[16] Zagadailov EA , Corman S , Chirikov V , et al. Real‐world effectiveness of brentuximab vedotin versus physicians' choice chemotherapy in patients with relapsed/refractory Hodgkin lymphoma following autologous stem cell transplantation in the United Kingdom and Germany. Leuk Lymphoma. 2018;59(6):1413‐1419. 29045163 10.1080/10428194.2017.1382698

[17] Badar T , Epperla N , Szabo A , et al. Trends in postrelapse survival in classic Hodgkin lymphoma patients after experiencing therapy failure following auto‐HCT. Blood Adv. 2020;4(1):47‐54. 31899797 10.1182/bloodadvances.2019000736 PMC6960457

[18] Bartlett NL , Chen R , Fanale MA , et al. Retreatment with brentuximab vedotin in patients with CD30‐positive hematologic malignancies. J Hematol Oncol. 2014;7:24. 24642247 10.1186/1756-8722-7-24 PMC3994656

[19] Fukuhara N , Yamamoto G , Tsujimura H , et al. Retreatment with brentuximab vedotin in patients with relapsed/refractory classical Hodgkin lymphoma or systemic anaplastic large‐cell lymphoma: a multicenter retrospective study. Leuk Lymphoma. 2020;61(1):176‐180. 31437057 10.1080/10428194.2019.1654100

[20] Sun J , Wang Y . Brentuximab vedotin: unexpectedly good response in CD30(−) mycosis fungoides. Br J Dermatol. 2019;180(6):1300‐1301. 31157447 10.1111/bjd.17726

[21] Jagadeesh D , Horwitz S , Bartlett NL , et al. Response to brentuximab vedotin by CD30 expression in non‐Hodgkin lymphoma. Oncologist. 2022;27(10):864‐873. 35948003 10.1093/oncolo/oyac137 PMC9526494

[22] Papadavid E , Kapniari E , Pappa V , et al. Multicentric EORTC retrospective study shows efficacy of brentuximab vedotin in patients who have mycosis fungoides and Sézary syndrome with variable CD30 positivity. Br J Dermatol. 2021;185(5):1035‐1044. 34137025 10.1111/bjd.20588

[23] Jacobsen ED , Sharman JP , Oki Y , et al. Brentuximab vedotin demonstrates objective responses in a phase 2 study of relapsed/refractory DLBCL with variable CD30 expression. Blood. 2015;125(9):1394‐1402. 25573987 10.1182/blood-2014-09-598763

[24] Onaindia A , Martínez N , Montes‐Moreno S , et al. CD30 expression by B and T cells: a frequent finding in angioimmunoblastic T‐cell lymphoma and peripheral T‐cell lymphoma‐not otherwise specified. Am J Surg Pathol. 2016;40(3):378‐385. 26574847 10.1097/PAS.0000000000000571

[25] Li F , Emmerton KK , Jonas M , et al. Intracellular released payload influences potency and bystander‐killing effects of antibody‐drug conjugates in preclinical models. Cancer Res. 2016;76(9):2710‐2719. 26921341 10.1158/0008-5472.CAN-15-1795

[26] Okeley NM , Miyamoto JB , Zhang X , et al. Intracellular activation of SGN‐35, a potent anti‐CD30 antibody‐drug conjugate. Clin Cancer Res. 2010;16(3):888‐897. 20086002 10.1158/1078-0432.CCR-09-2069

[27] Müller P , Martin K , Theurich S , et al. Microtubule‐depolymerizing agents used in antibody‐drug conjugates induce antitumor immunity by stimulation of dendritic cells. Cancer Immunol Res. 2014;2(8):741‐755. 24916470 10.1158/2326-6066.CIR-13-0198

